# Changes in the Prevalence of Infection in Pregnant Women during the COVID-19 Lockdown

**DOI:** 10.3390/microorganisms11081973

**Published:** 2023-07-31

**Authors:** Dominique E. Werter, Heleen J. Schuster, Caroline Schneeberger, Eva Pajkrt, Christianne J. M. de Groot, Elisabeth van Leeuwen, Brenda M. Kazemier

**Affiliations:** 1 Department of Obstetrics and Gynaecology, Amsterdam UMC Location University of Amsterdam, Meibergdreef 9, 1105 AZ Amsterdam, The Netherlands; 2 Amsterdam Reproduction and Development Research Institute, 1105 AZ Amsterdam, The Netherlands; 3 Department of Medical Microbiology and Infection Control, Amsterdam UMC Location University of Amsterdam, Meibergdreef 9, 1105 AZ Amsterdam, The Netherlands; 4 National Institute for Public Health and the Environment (RIVM), Centre for Infectious Disease Control, 3721 MA Bilthoven, The Netherlands; 5 Department of Human Genetics, Amsterdam UMC Location Vrije Universiteit Amsterdam, De Boelelaan 1117, 1081 HV Amsterdam, The Netherlands; 6 Department of Obstetrics, Division Women and Baby, Birth Centre Wilhelmina’s Children Hospital, University Medical Center Utrecht, 3584 CX Utrecht, The Netherlands

**Keywords:** pregnancy, infections, COVID-19 lockdown, *group B-Streptococcus*, cytomegalovirus, urinary tract infections

## Abstract

Background: During the outbreak of SARS-CoV-2, strict mitigation measures and national lockdowns were implemented. Our objective was to investigate to what extent the prevalence of some infections in pregnancy was altered during different periods of the COVID-19 pandemic. Methods: This was a single centre retrospective cohort study conducted in the Netherlands on data collected from electronic patient files of pregnant women from January 2017 to February 2021. We identified three time periods with different strictness of mitigation measures: the first and second lockdown were relatively strict; the inter-lockdown period was less strict. The prevalence of the different infections (Group B *Streptococcus* (GBS)-carriage, urinary tract infections and Cytomegalovirus infection) during the lockdown was compared to the same time periods in previous years (2017–2019). Results: In the first lockdown, there was a significant decrease in GBS-carriage (19.5% in 2017–2019 vs. 9.1% in 2020; *p* = 0.02). In the period following the first lockdown and during the second, no differences in prevalence were found. There was a trend towards an increase in positive Cytomegalovirus IgM during the inter-lockdown period (4.9% in 2017–2019 vs. 12.8% in 2020; *p* = 0.09), but this did not reach statistical significance. The number of positive urine cultures did not significantly change during the study period. Conclusions: During the first lockdown there was a reduction in GBS-carriage; further studies are warranted to look into the reason why.

## 1. Introduction

From early 2020 to May 2023, the world faced a pandemic caused by the SARS-CoV-2 virus: Coronavirus Disease 2019 (COVID-19). Recent studies focused on prognostic markers, interventions and treatment options to better understand the disease and reduce its impact [1,2,3]. On the other side, strict mitigation measures and national lockdowns were implemented to prevent the spread of SARS-CoV-2. In addition, lockdowns (confinement), physical distancing, regular hand washing, and facemask wearing were introduced to prevent the spread.

Besides limiting the spread of SARS-CoV-2, it was shown that the lockdown had other effects: a decrease in preterm births, increase in stillbirths and a higher rate of maternal depression [4,5,6,7]. Preterm birth was thought to be the result of a complex pathway with different underlying mechanisms such as cervical insufficiency and infection (e.g., urinary tract infections (UTIs) [8] and *Streptococcus agalactiae* also known as Group B *Streptococcus* (GBS)-carriage) [9,10]. For decades researchers had been trying to reduce the number of preterm births, but no intervention showed as great an effect as the recent lockdown for COVID-19. This showed that the number of preterm births was more modifiable than previously thought and could perhaps be influenced by changes in behaviour during pregnancy.

In addition, infections can have profound effects on pregnancy and the neonate, such as sensorineural deafness from the cytomegalovirus (CMV) or hydrocephalus from toxoplasmosis [11,12,13,14]. Maternal GBS-carriage could lead to early-onset neonatal sepsis, which is an important cause of neonatal mortality [9]. Infection in pregnancy is therefore an important topic of research.

Previous research showed that mitigation measures not only slowed the spread of COVID-19, but also of other infections. During the lockdown, there was a decrease in prevalence or change in the (seasonal) pattern of respiratory syncytial virus and parainfluenza [15,16]. But there may also have been an effect on less-obvious infections. For example, hygiene measures were proven to be effective in reducing the prevalence of UTIs in pregnancy [17]. Due to reduced interactions among people during lockdowns, possibly including reduced sexual interaction and altered hygiene, other infectious processes may have been affected as well. Whether the increased focus on hygiene also influenced the prevalence of infections in pregnancy is unknown.

Since the mitigation measures may have had an effect on the prevalence of infections with consequences for pregnancy outcomes, we performed this study to investigate to what extent the prevalence of some infections in pregnancy was altered during the lockdown.

## 2. Material and Methods

### 2.1. Study Design

We performed a single-centre retrospective cohort study based on data collected from electronic patient files of pregnant women in the Amsterdam UMC, location VUmc, between January 2017 and February 2021. The VUmc is a tertiary referral centre in Amsterdam, the Netherlands.

### 2.2. Inclusion

Pseudonymized care data on pregnancy and laboratory results related to infections and prescribed antibiotics were extracted from the patient files. Women were included if they had not had any laboratory recording or antibiotic prescriptions during their active pregnancy DOT (“Diagnose Behandel Combinatie Op weg naar Transparantie”). DOT is a system in the Netherlands used to describe the costs for a complete care path, based on the diagnosis in this case pregnancy or birth, when a patient receives care in a hospital.

Laboratory results from blood, urine, and rectovaginal swabs were collected from patients admitted to the clinic or from those visiting the outpatient clinic. All women who delivered in the VUmc during the years 2017–2021 were included.

### 2.3. Definitions

The data collection was pseudonymized, so no clinical data, estimated date of delivery, symptoms or signs of infection were available.

Throughout 2020 and 2021, different measures were taken to limit the spread of COVID-19 in the Netherlands. Based on the strictness of these measures, we identified three time periods. The first period, “first lockdown”, started on 12 March 2020. From that moment on, people were advised to work from home and avoid contact with vulnerable people. In the week thereafter, schools, restaurants, bars and non-essential services that involved physical contact were shut down. We defined the end of the first lockdown period to be 1 June 2020, when primary schools, bars and restaurants re-opened and high schools partially reopened (Table 1) [18].

The second period, “inter-lockdown”, with few mitigation measures, was defined from 1 June to 14 October 2020, on which date a second (partial) lockdown was implemented in the Netherlands (Table 1) [18]. On 8 February 2021, primary schools reopened [18]. Therefore, the third period, “second lockdown” was defined from 14 October 2020 to 8 February 2021 (Table 1).

We compared the prevalence of infections during the lockdowns and inter-lockdown to the same calendar days in the years 2017–2019 (pre-COVID-19) to avoid seasonal effects.

In the Netherlands, a risk factor-based screening for GBS-carriage took place. Screening for GBS-carriage was recommended in case of a preterm birth or prelabour-ruptured membranes for more than 24 h, and had to be discussed in case of GBS-carriage in a previous pregnancy or when a previous neonate had an early onset sepsis or meningitis with an unknown cause. In the last two cases, screening had to take place between 35 and 37 weeks of gestational age. A rectovaginal swab was used for screening [12].

It is also common practice in the Netherlands to check for GBS-carriage if a urine culture is performed for a suspected UTI in pregnant women. Since the indication for routine rectovaginal screening in patients is different from an incidental finding of GBS for a UTI complaint, we decided to use only the rectovaginal swabs to check for GBS-carriage, which was defined by the first (recto)vaginal swab in which GBS was cultured in case more than one swab was taken in a period. Prevalence of GBS-carriage was calculated as the number of women with a positive GBS divided by the number of women in whom GBS-carriage was tested over the same time period.

In the Netherlands, a urine culture is only performed when a pregnant woman complains of a UTI (e.g., frequent urination, preterm contractions) and in some cases after completion of antibiotics for a UTI [19]. We considered a urine culture positive when there were 1 or 2 bacterial species present with ≥10^5^ colony-forming units. We only calculated the first positive urine culture per lockdown period since it was not always clear if women had been treated with antibiotics in the meantime. Prevalence of a UTI was calculated as the number of pregnant women with a UTI divided by the number of pregnant women in whom urine was cultured over the same time period.

CMV is not routinely screened during pregnancy in the Netherlands, but sometimes pseudoscreening is done in high-risk women such as healthcare workers and teachers. In addition, CMV can be tested during pregnancy when there is a clinical suspicion of a possible CMV infection based on ultrasound anomalies (such as ventriculomegaly and foetal growth restriction), or if the woman had come in contact with someone known to be CMV positive [20]. The definition we used for a CMV infection was the first positive CMV IgM in serum during pregnancy. All doubtful results (with test results around the cut-off) were considered negative since we had no clinical data. For the purpose of this study, we calculated the prevalence of CMV as the number of pregnant women with a first positive CMV IgM divided by all pregnant women in whom CMV IgM was tested over the same lockdown period.

*Chlamydia* in pregnancy is tested only in specific situations, e.g., repetitive vaginal blood loss, unusual vaginal discharge and being HIV-positive. The definition we used for a positive *Chlamydia* is a positive *Chlamydia trachomatis* PCR in a cervicovaginal swab. The prevalence of *Chlamydia* was calculated as the number of women with a positive test divided by the number of women in whom the same test was performed.

In the Netherlands, parvovirus-B19 is only tested in specific situations: in certain high-risk groups such as primary school teachers, or when foetal growth restriction or hydrops fetalis occurs [14]. The definition for parvovirus-B19 was a positive serum IgM for parvovirus-B19 in pregnancy. The prevalence of parvovirus-B19 was calculated as the number of women with a positive test divided by the number in whom the same test was performed.

Toxoplasmosis was tested when the need for a test was indicated, like hydrocephalus or foetal growth restriction [13]. The determination for toxoplasmosis was a positive serum *Toxoplasmosis gondii* IgM in pregnancy, and its prevalence was calculated as the number of women with a positive test divided by the number in whom the same test was performed.

Since there were limited results on *Chlamydia,* parvovirus-B19 and toxoplasmosis, we divided these cohorts in two time periods pre-lockdown (1 January 2017 to 12 March 2020) and during the lockdown (12 March 2020 to the end of February 2021) for comparison.

### 2.4. Statistical Analysis

Since some infections were quite rare, we included only laboratory tests when there were at least two positive results per year (e.g., HIV and rubella were excluded). After this selection we continued with GBS-carriage, UTI, CMV, *Chlamydia*, toxoplasmosis and parvovirus-B19 infection. To analyse the influence of the different lockdown time periods, we selected infections that had at least 10 positive cases in 2020.

The prevalence between the different time periods was compared using the Fisher exact test in case of <10 cases, or a Chi-Square test if there were ≥10 cases. The percentage of tests per number of women that delivered in the hospital per year was compared using One-way ANOVA. The prevalence of *Chlamydia*, toxoplasmosis and parvovirus-B19 infection pre-lockdown and during lockdown was compared using the Fisher exact test.

A *p* value < 0.05 was considered statistically significant.

In addition, we investigated whether the number of collected samples changed over the years. We divided the number of collected samples by the number of women who delivered over the same period in our hospital to correct for possible differences in the number of women who visited.

We used IBM SPSS statistics 28 for statistical analyses.

### 2.5. Ethical Approval

The Medical Research Involving Human Subjects Act does not apply to this study, an official approval of this study by the Medical Ethics Review Committee of the Academic Medical Centre was therefore not required (reference number W21_475 # 21.527app).

## 3. Results

We included 234,223 laboratory results that had been collected from patients between 1 January 2017 and 1 March 2021. After excluding incomplete data, 1788 rectovaginal swabs for GBS were included. There were 3223 urine cultures from 2256 pregnant women and 771 CMV IgM results from 416 women.

### 3.1. GBS

During the first lockdown, we noted a statistically significant decrease in GBS-positive women (19.7% in 2017–2019 vs. 8.6% in 2020; *p* = 0.01, Table 2). However, in the inter-lockdown and second lockdown period, the number of GBS carriers remained stable, which conformed to the same period before the first lockdown (respectively 21.7% in 2017–2019 vs. 18.4% in 2020; *p* = 0.44 in the inter-lockdown and 20.4% in 2017–2019 vs. 20.0% in 2020 in the second lockdown; *p* = 0.91, Table 2).

### 3.2. Urine Cultures

During the first lockdown period there was no statistically significant difference in positive urine cultures (7.5% in 2017–2019 vs. 5.0% in 2020; *p* = 0.61, Table 2). In the two following time periods there was a non-statistically significant increase of positive urine cultures (respectively 6.9% in 2017–2019 vs. 9.2% in 2020; *p* = 0.35 and 6.8% in 2017–2019 vs. 11.6% in 2020; *p* = 0.09, Table 2).

### 3.3. CMV

During the first lockdown we saw no statistically significant difference in the number of women with a positive CMV IgM (6.3% in 2017–2019 vs. 4.3% in 2020; *p* = 0.72, Table 2). In the following inter-lockdown period, we saw a trend towards more CMV IgM positive women but this did not reach statistical significance (4.9% in 2017–2019 vs. 12.8% in 2020; *p* = 0.09, Table 2). During the second lockdown there was no statistically significant difference (13.5% in 2017–2019 vs. 8.6% in 2020; *p* = 0.43, Table 2).

**Figure 1:**
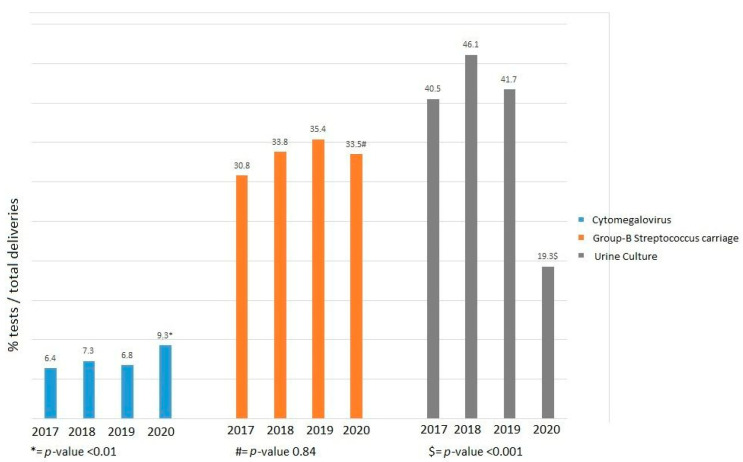
Percentage of tests per number of women that delivered in the hospital per year.

There was no statistically significant difference in the percentage of GBS swabs collected divided by the total deliveries over the different years (all between 31–35%; *p* = 0.84, Figure 1). The percentage of samples taken to detect a UTI over total deliveries statistically significantly decreased in 2020 compared to the previous years (2017–2019 40.5–46.1% vs. 2020 19.3%; *p* < 0.001, Figure 1). In 2020 there was a statistically significant increase of CMV IgM samples taken compared to 2017–2019 (2017–2019 6.4–7.3% vs. 2020 9.3%; *p* < 0.01, Figure 1). The total number of deliveries decreased slightly over the years: 1659 deliveries in 2017, 1498 in 2018, 1431 in 2019, 1326 in 2020 and 1326 in 2021.

### 3.4. Other Infections

**Figure 2:**
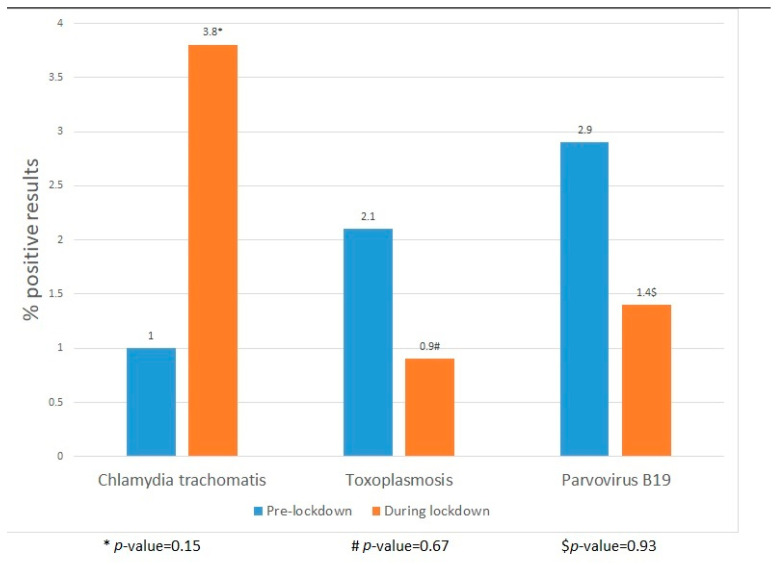
Positive chlamydia, toxoplasmosis and parvovirus-B19 pre-lockdown and during the lockdown.

The number of positive *Chlamydia*, toxoplasmosis and parvovirus-B19 infections did not significantly differ between 2017–2019 and 2020 (*Chlamydia* 2/195 vs. 3/80, toxoplasmosis 5/241 vs. 1/117 and parvovirus-B19 4/140 vs. 1/69, Figure 2).

## 4. Discussion

### 4.1. Main Findings

We found a significant decrease in women with a positive GBS swab during the first lockdown but not during the second compared to the same time period in previous years. Maternal GBS-carriage is associated with early onset neonatal sepsis and with preterm birth [9,12]. Until now, it was unclear whether GBS-carriage could be influenced by lifestyle or hygiene measures. The decrease in this study could be a starting point for investigating how to reduce GBS-carriage in pregnant women.

The number of women with a UTI did not significantly change; however, a reduction in the number of cultures collected was noted from the first lockdown onwards. This underlines what had been previously found: patients visited hospitals less during the COVID-19 pandemic.

Finally, we found a trend towards an increasing number of pregnant women with a positive CMV IgM in the inter-lockdown period compared to the same time period in previous years, but this did not reach statistical significance. The increased number of positive CMV IgM is possibly because schools reopened. Women who had more than one child were more exposed to CMV.

### 4.2. Strengths and Limitations

To our knowledge, this is the first study reporting on the prevalence of infections in pregnancy during lockdown for COVID-19. A strength of this study is that it compared the prevalence of infections during the lockdown with the prevalence during the same time period in preceding years. In this way, we avoided any possible seasonal effect of infections and pregnancy. Unfortunately, we had no access to any clinical data, therefore the indication for testing was not known, and neither were we able to distinguish between a clinical or subclinical infection and an non-specific reaction. In addition, correcting for confounders was not possible. However, over different time periods we used the same definitions thereby limiting the impact on our outcomes.

Another limitation was that the sample size was small and that this was a single centre study. We cannot exclude to possibility that this resulted in a regression to the mean. In addition, we did not have the exact gestational age of the pregnancy during the infection. Although this did not have any implications for vulnerability to infections, it would have strengthened clinical implications. Unfortunately, we did not have information for when the GBS screening was performed, the elective screening around 35–37 weeks of gestational age, or for an indication of a possible preterm birth or broken membranes for more than 24 h.

### 4.3. Interpretation

About 18% of pregnant women were colonized with GBS. Transmission often took place vertically, from mother to child during the delivery [21]. GBS is a commensal bacterium in the gastrointestinal tract [22]. One study in the general population showed that there was an association between handwashing and vaginal colonization of GBS [23], which is transmissible through oral–fecal and anal–vaginal routes in the general population [22,23]. With increased awareness of infections, we hypothesize that people would wash their hands better also after toilet use thereby lowering transmission via the oral-faecal route. This could explain why we found a significant decrease in GBS in pregnant women during the first lockdown. The fact that the rate of GBS in the inter-lockdown and the second lockdown periods was unchanged can be explained by the fact that despite high rates of COVID-19 infection, adherence to safety measures in the second lockdown was much lower compared to that of the first lockdown [24].

Indications to test for GBS did not change during different lockdowns, which is why we expected a reliable sample, not an over- or underestimation, of GBS-carriage. This was supported by the fact that we did not see a decrease or increase in GBS samples taken over the years.

We found a higher prevalence of CMV in our cohort (4.3–13.5%) compared to the general literature (3–4.1%) [20]. The reason for this is that CMV was only tested if there were a suspected infection or fetal growth restriction as opposed to screening the whole population. Also, the samples were from a tertiary referral centre meaning from a high-risk population. In addition, we defined positive IgM as a positive CMV infection, even though a CMV-PCR on amniotic fluid or neonatal blood or urine is the gold standard for diagnosing a congenital CMV infection. CMV IgM could stay positive up for to a few months or be a false positive, which could also explain the high positivity rates. Since there was no policy change over the past five years regarding the indication to test for CMV, and we used the same definition throughout the years, we did think that the lockdown affected CMV transmission. However, our study was not large enough to show a statistically significant difference. Our hypothesis was that there was initially a decrease in CMV infections in the first lockdown period followed by an increase in CMV in the inter-lockdown. The transmission route of CMV was through body fluids like saliva and urine, most frequently transmitted by close contact with young children [25]. The reason for our findings could be that the closing and re-opening of schools and day-care centres, went hand in hand with, respectively, a lower and high exposure of women to CM-infected children. The fact that the lockdown affected CMV prevalence in pregnancy was underlined by another study that found a significant decrease in the number of CMV positive saliva swabs in neonates during the lockdown [26].

We saw a slight increase in the number of samples taken for CMV in 2020 in our study compared to other years. CMV was only collected in case of a suspected infection either from ultrasound anomalies or contact with a known CMV-positive individual.

The most evident result concerning the urine cultures is the fact that significantly fewer urine cultures were taken during 2020. Our hypothesis is that fewer pregnant women visited the hospital to check for UTIs during the pandemic. Especially during the first lockdown, people were afraid to go to the hospital because of the fear of being infected with SARS-CoV-2 [27,28]. This fear led to a reduction in planned hospital visits in pregnant women, but also fewer emergency visits and non-urgent emergency-department visits [6,29,30]. In the Netherlands the majority of pregnant women with a UTI visited a general practitioner instead of going to the hospital. It was shown that even though the total number of patients presenting themselves to a care giver decreased, they went more often to their general practitioner’s practice than the hospital [30,31]. In addition, fewer people were referred for medical care. Even after a referral, people would not go to a hospital [32,33]. We therefore hypothesize that during the lockdown, care for UTI probably was shifted to the general practitioner instead of the hospital. However, we do not have data to support this hypothesis. In addition, we do not have evidence that hygiene measures reduced the risk for UTIs.

## 5. Conclusions

Our data showed that the prevalence of GBS-carriage was affected during the first lockdown. These results could suggest that GBS-carriage was more modifiable in pregnancy than previously thought. Unfortunately, we do not know which specific measure or action during the first lockdown led to a decrease in the prevalence of GBS-carriage in pregnant women. Future studies should determine if there is a hygiene measure or lifestyle change that is able to decrease the prevalence of GBS-carriage. Hopefully we will find an easy-to-implement hygiene recommendation for pregnant women that can reduce GBS-carriage and GBS sepsis in subsequent neonates.

## Figures and Tables

**Table 1:** Mitigation measures during the COVID-19 pandemic from March 2020 to February 2021.

	Date	Measures
**First lockdown**	12 March 2020	- Strict advice to limit contact with vulnerable people - People working in healthcare and vital sectors stayed at home when experiencing fever or complaints - Other people worked from home as much as possible
	15 March 2020	Closing of bars and restaurants
	16 March 2020	- It was forbidden to practice a profession in which physical contact was necessary such as hairdressers (except (para-)medical professions) - Closing of schools and daycares
	23 March 2020	- Maximum of three visitors at home - No gatherings allowed - At all locations 1.5 m distance had to be maintained
	11 May 2020	- Most professions in which physical contact was necessary were allowed to operate again - Primary schools opened for half-day classes, daycares opened again
**Inter-lockdown**	1 June 2020	- High schools open for 25% of the time - Bars and restaurants open again
	8 June 2020	- Primary schools opened 100%
	1 July 2020	- No limitations to the number of people at gatherings - Maintaining 1.5 m distance for adults, not for children
	6 August 2020	- All introductory activities for the upcoming school years are online
	18 August 2020	- Gatherings of a maximum of six people at home with social distancing
	29 September 2020	- Gatherings of a maximum of three people at home with social distancing - Bars and restaurants have to close at 22:00 - Compulsory registration for all people visiting a professional where social distancing was not possible - Amateur sport matches without public - Closing of sports canteens
**Second lockdown**	14 October 2020	- Closing of bars and restaurants - Hosting events forbidden - Compulsory facemasks in higher education facilities - Limitation of maximum four people doing sports together with social distancing, matches are not allowed anymore
	4 November 2020	- Strict advice to stay home - Maximum of two visitors, or one household at home per day, with social distancing - Public spaces closed - Limitation of maximum two people doing sports together with social distancing except for children and professional players
	14 December 2020	- Closing of non-essential shops - Closing of gyms - Schools deliver distance education - Closing of daycares
	Christmas 2020	- Maximum of 3 visitors at home
	20 January 2021	- Maximum of 1 visitor at home
	23 January 2021	- Curfew between 21:00 and 4:30
	8 February 2021	- Opening of primary schools and daycares - Picking up goods from non-essential shops allowed with appointment

Adapted from https://www.rivm.nl/gedragsonderzoek/tijdlijn-maatregelen-covid (accessed on 21 March 2022) [18].

**Table 2:** Positive GBS swabs, urine cultures and CMV IgM during lockdowns for COVID 19.

GBS		2017	2018	2019	2020–2021 ^#^	*p*-Value *
First Lockdown	%	22.0	18.5	18.8	8.6	0.01
12 March until 1 June	N/N total	27/123	24/130	22/117	8/93	
Inter-lockdown	%	25.0	21.1	19.3	18.4	0.44
1 June until 14 October	N/N total	42/168	38/180	36/187	29/158	
Second Lockdown	%	19.0	17.6	24.1	20.0	0.91
14 October until 8 February	N/N total	22/116	27/153	38/158	41/205	
**Urine cultures**						
First Lockdown	%	5.8	5.7	10.0	5.0	0.61
12 March until 1 June	N/N total	10/171	11/192	20/200	3/60	
Inter-lockdown	%	8.8	6.7	6.8	9.2	0.35
1 June until 14 October	N/N total	20/288	19/284	14/207	11/119	
Second Lockdown	%	6.5	4.1	10.3	11.6	0.09
14 October until 8 February	N/N total	11/170	10/241	21/203	14/121	
**CMV IgM**						
First Lockdown	%	3.7	14.3	0	4.3	0.72
12 March until 1 June	N/N total	1/27	4/28	0/24	1/23	
Inter-lockdown	%	7.3	2.4	5.0	12.8	0.09
1 June until 14 October	N/N total	3/41	1/42	2/40	6/47	
Second Lockdown	%	25.0	6.9	13.8	8.6	0.43
14 October until 8 February	N/N total	4/16	2/29	4/29	6/70	
Total number of deliveries	N	1659	1498	1431	1326 ^$^	

* we compared 2017–2019 to 2020–2021. ^#^ 2021 was only used in the second lockdown period since the period exceeds the calender year. ^$^ only 2020. N: is number of women with infection. N total: is total of women tested for the infection.

## Data Availability

Upon reasonable request individual participant data is available. This will be all of the individual participant data collected during this, after de-identification. The waiver from the medical ethical commission is available. All data will be available after three months of publication. For fifteen years. We will share the data with researchers who provide a methodologically sound proposal. Data will be available for individual participant data meta-analysis. Proposals should be directed to d.e.werter@amsterdamumc.nl to gain access.
